# Supplementary figures and images for: Mcm2 promotes stem cell differentiation via its ability to bind H3-H4
Source: eLife. 2022 Nov 10;11:e80917. doi: 10.7554/eLife.80917 (PMC9681210; doi:10.7554/eLife.80917)

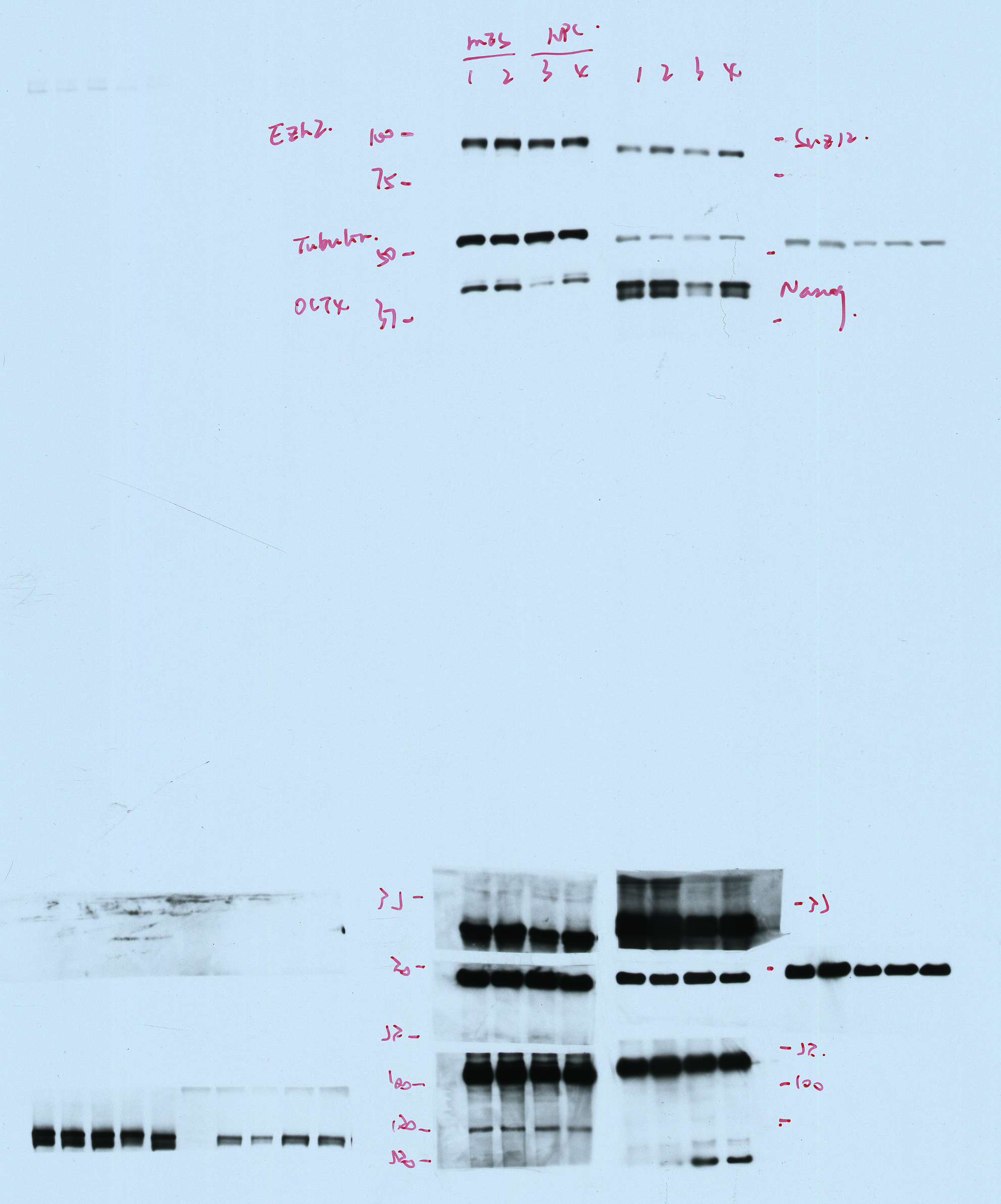

Supplement: Figure 2—source data 2. — Pou5f1 and Nanog protein levels in wild type (WT) and Mcm2-2A embryonic stem cells (ESCs) and neural precursor cells (NPCs). [file elife-80917-fig2-data2.zip › Figure2-source data2-full blot.tif]

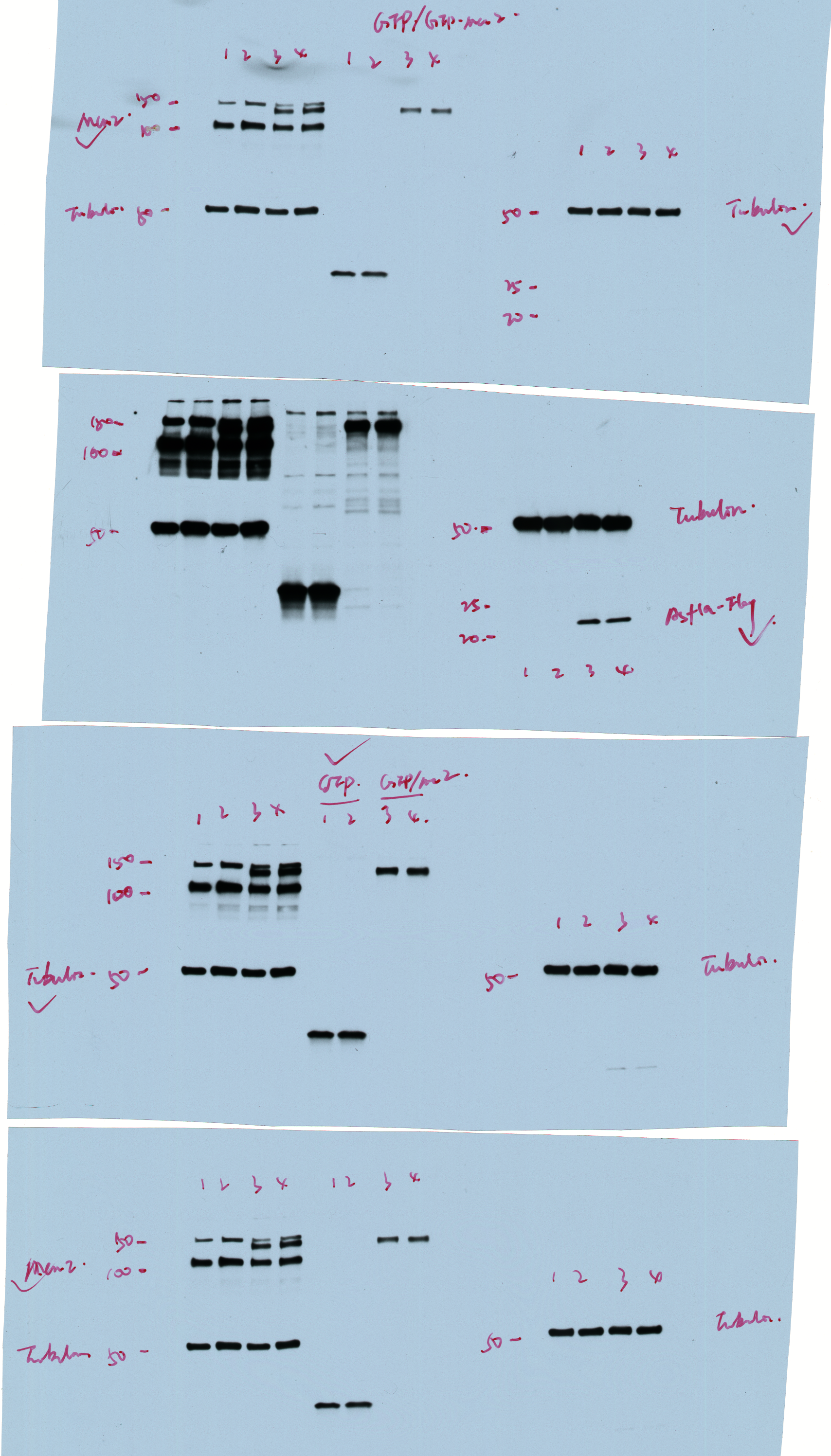

Supplement: Figure 2—source data 3. — pWPXL and pWPXL-Mcm2 expression in wild type (WT) and Mcm2-2A embryonic stem (ES) cells. [file elife-80917-fig2-data3.zip › Figure 2-source data 3-full plot.tif]

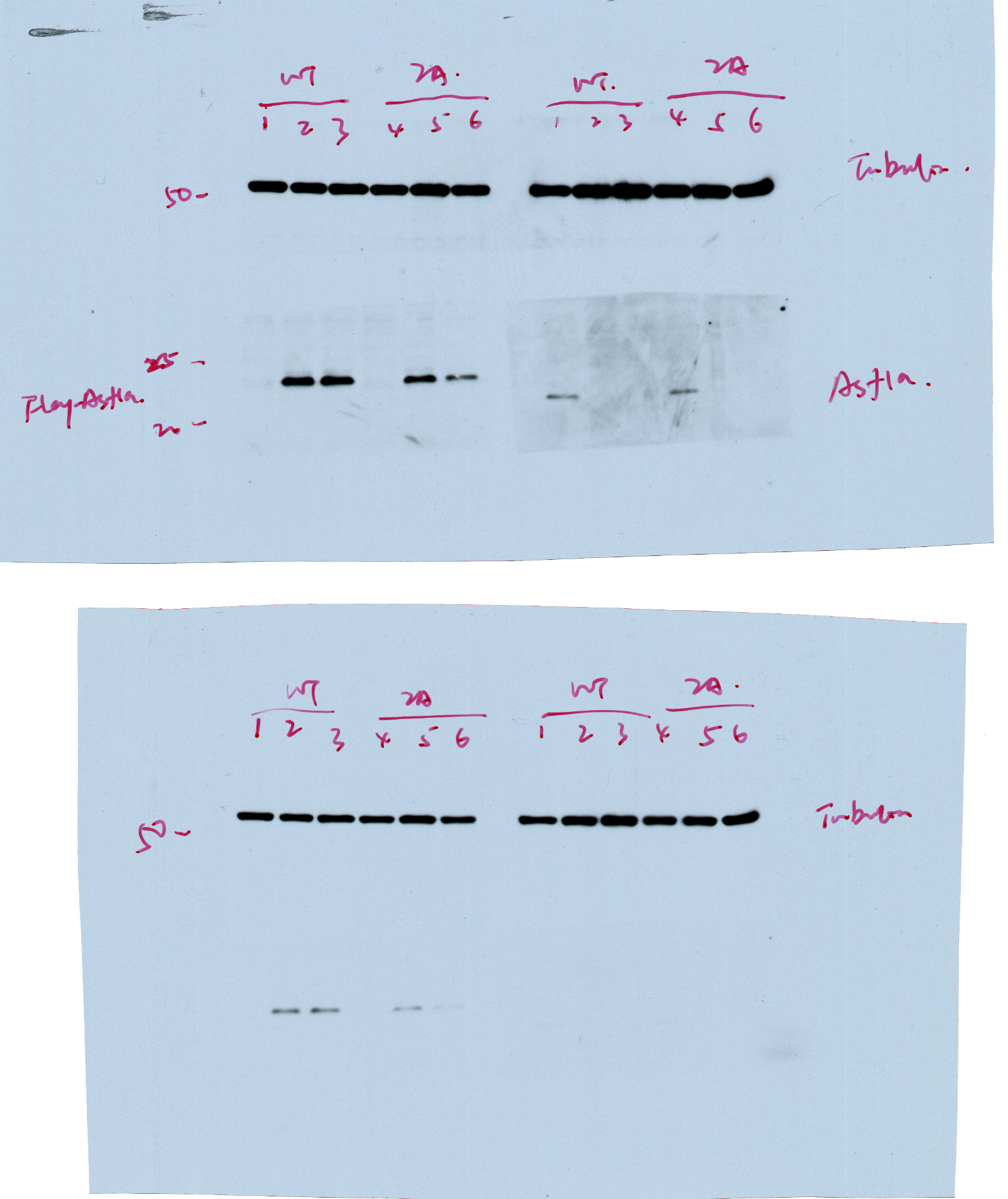

Supplement: Figure 2—figure supplement 2—source data 1. — pWPXL-Asf1a expression and Asf1a KO detection in wild type (WT) and Mcm2-2A embryonic stem (ES) cells. [file elife-80917-fig2-figsupp2-data1.zip › Figure 2-figure supplement 2-source data 1-full plot2.tif]

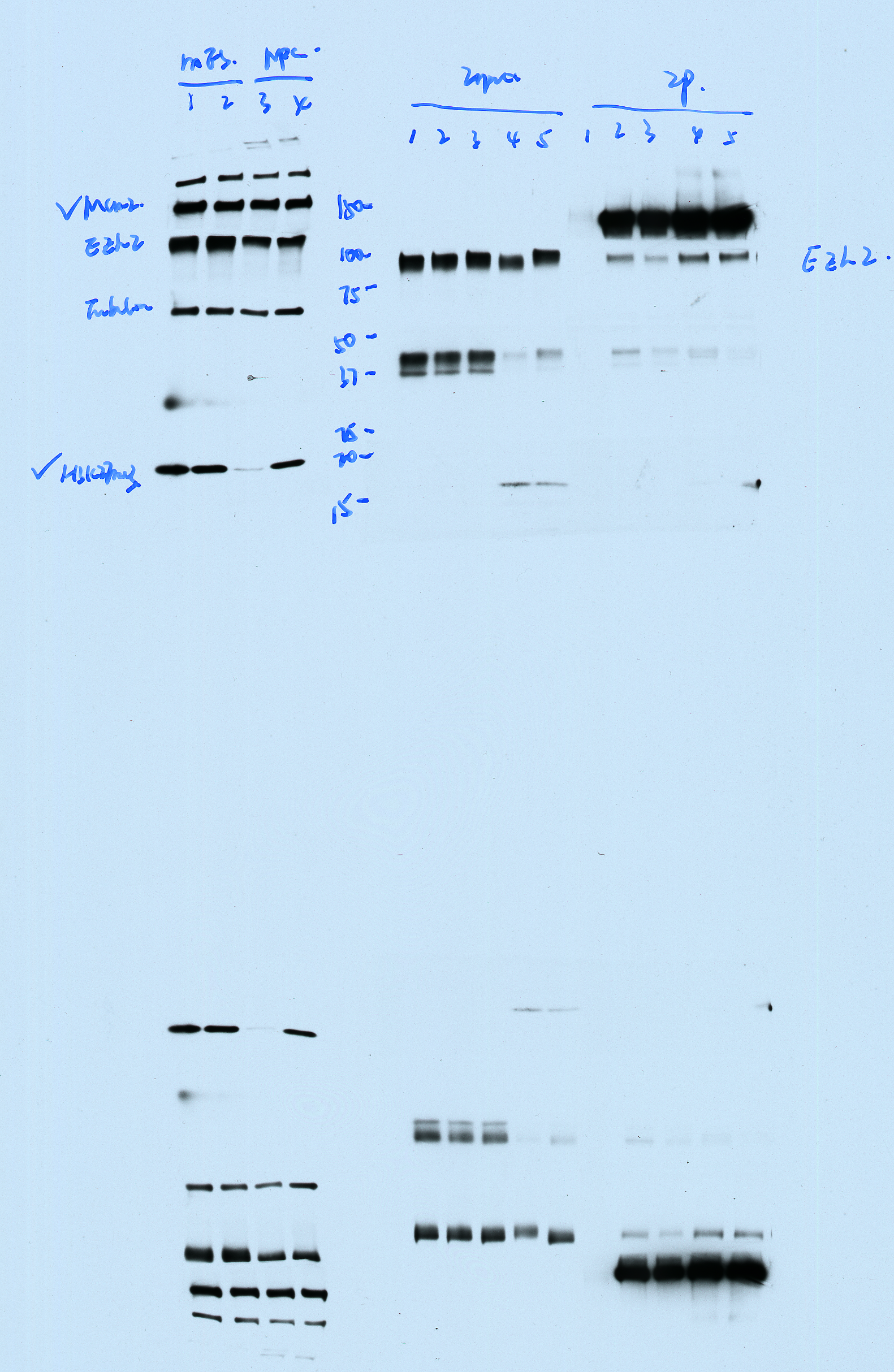

Supplement: Figure 4—source data 1. — H3K4me3, H3K27me3, Ezh2, Mcm2, Pou5f1, and Nanog protein levels in wild type (WT) and Mcm2-2A embryonic stem cells (ESCs) and neural precursor cells (NPCs). [file elife-80917-fig4-data1.zip › Figure4-source data1-full blot1.tif]

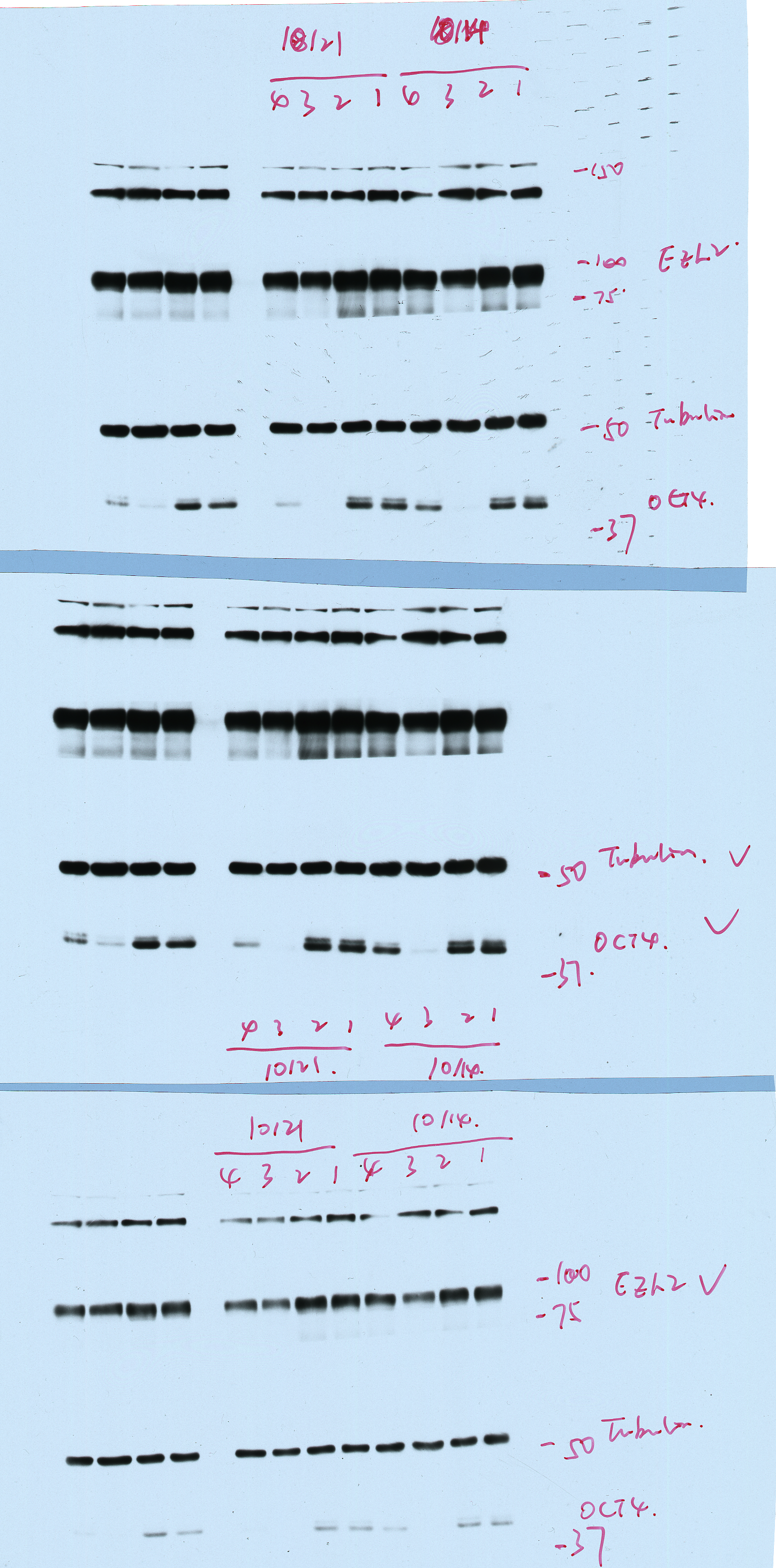

Supplement: Figure 4—source data 1. — H3K4me3, H3K27me3, Ezh2, Mcm2, Pou5f1, and Nanog protein levels in wild type (WT) and Mcm2-2A embryonic stem cells (ESCs) and neural precursor cells (NPCs). [file elife-80917-fig4-data1.zip › Figure4-source data1-full blot2.tif]

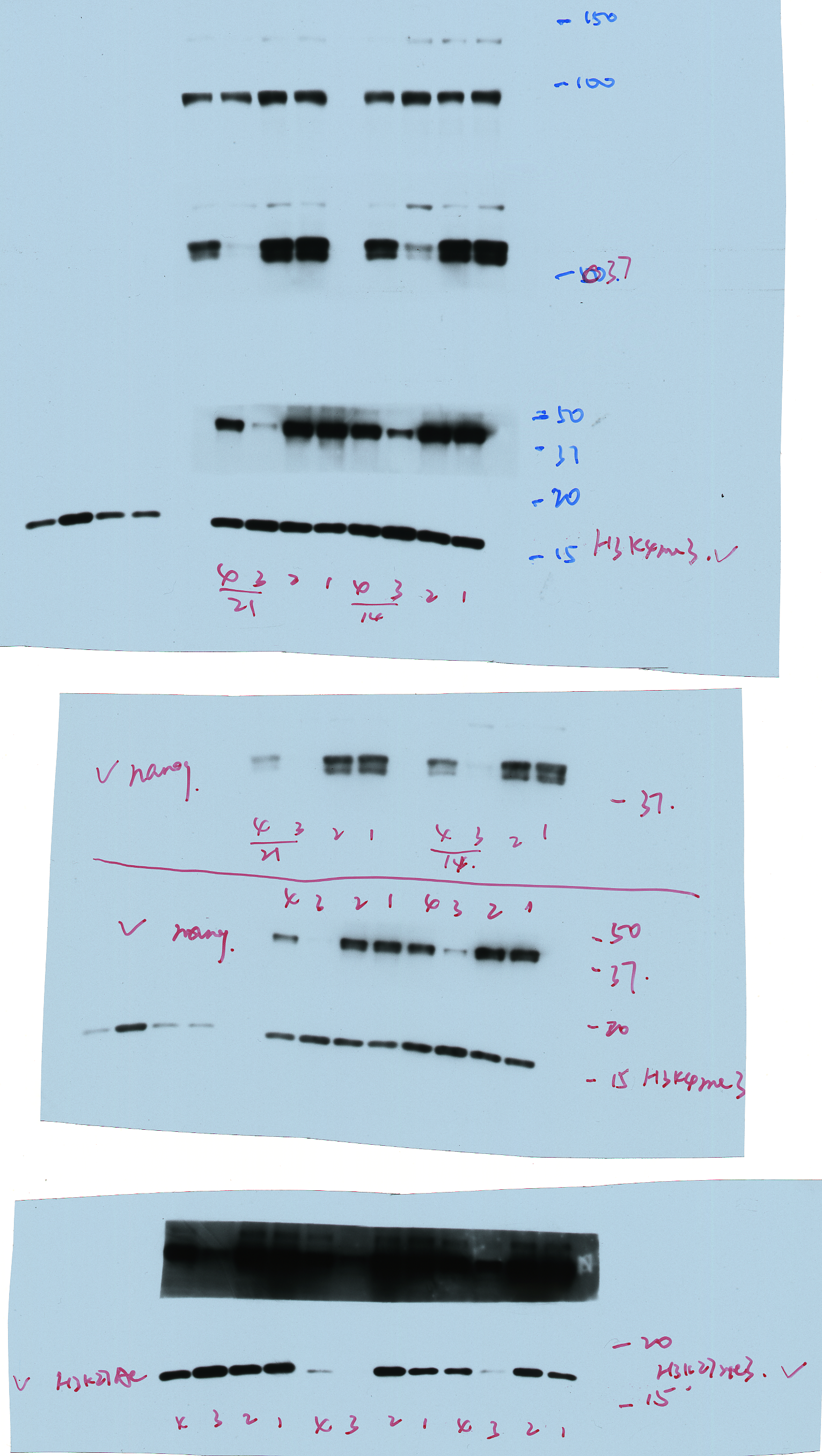

Supplement: Figure 4—source data 1. — H3K4me3, H3K27me3, Ezh2, Mcm2, Pou5f1, and Nanog protein levels in wild type (WT) and Mcm2-2A embryonic stem cells (ESCs) and neural precursor cells (NPCs). [file elife-80917-fig4-data1.zip › Figure4-source data1-full blot3.tif]
